# Soluble MD-2 and Heme in Sickle Cell Disease Plasma Promote Pro-Inflammatory Signaling in Endothelial Cells

**DOI:** 10.3389/fimmu.2021.632709

**Published:** 2021-03-26

**Authors:** Ping Zhang, Julia Nguyen, Fuad Abdulla, Alexander T. Nelson, Joan D. Beckman, Gregory M. Vercellotti, John D. Belcher

**Affiliations:** ^1^ Division of Hematology, Oncology and Transplantation, Department of Medicine, University of Minnesota, Minneapolis, MN, United States; ^2^ University of Minnesota School of Medicine, Minneapolis, MN, United States

**Keywords:** soluble MD-2, sickle cell disease, IL-8, endothelial cell, TLR4, heme, hemopexin, IL-6

## Abstract

Recent evidence indicates that hemolysis in sickle cell disease (SCD) promotes inflammation *via* innate immune signaling through toll-like receptor 4 (TLR4). Free heme released by hemolyzed red blood cells can bind to myeloid differentiation factor-2 (MD-2) and activate TLR4 pro-inflammatory signaling on endothelium to promote vaso-occlusion and acute chest syndrome in murine models of SCD. MD-2 is co-expressed with TLR4 on cell membranes, but in inflammatory conditions, soluble MD-2 (sMD-2) is elevated in plasma. sMD-2 levels were significantly increased in human and murine sickle (SS) plasma as compared to normal (AA) plasma. Human umbilical vein endothelial cells (HUVEC) and human lung microvascular endothelial cells incubated with human SS plasma had significant increases in pro-inflammatory IL-8, IL-6, and soluble VCAM-1 secretion compared to endothelial cells incubated with AA plasma. The increase in HUVEC IL-8 secretion was blocked by depletion of sMD-2 from SS plasma and enhanced by the addition of sMD-2 to AA plasma. The TLR4 signaling inhibitor, TAK-242, inhibited HUVEC IL-8 secretion in response to SS plasma by 85%. Heme-agarose pull-down assays and UV/Vis spectroscopy demonstrated that heme binds to sMD-2. Hemopexin, a high affinity heme-binding protein, inhibited HUVEC IL-8 secretion induced by SS plasma or SS and AA plasma supplemented with sMD-2. These data suggest that sMD-2 bound to heme might play an important role in pro-inflammatory signaling by endothelium in SCD.

## Introduction

Sickle cell disease (SCD) is caused by a single point mutation (Glu->Val) at position 6 in the β-globin gene that leads to polymerization of deoxy-hemoglobin S (HbS) and the characteristic sickling of red blood cells. The ongoing polymerization of HbS promotes hemolysis, inflammation, and vaso-occlusive pain crises (1). During hemolysis, HbS is released into the vasculature and readily oxidized to methemoglobin, which can release free heme (2, 3). Normally, free hemoglobin and heme in plasma are safely cleared by haptoglobin and hemopexin (4, 5). However in SCD, chronic hemolysis depletes circulating haptoglobin and hemopexin (6, 7), allowing free heme to activate toll-like receptor 4 (TLR4) signaling on endothelial and inflammatory cells (3, 8–10). TLR4 signaling on endothelium leads to NF-ĸB activation, rapid release of Weibel-Palade bodies, discharge of P-selectin and von Willebrand factor, and vaso-occlusion in murine models of SCD (3).

TLR4 signaling induced by heme or its canonical ligand, lipopolysaccharide (LPS), requires cofactors CD14 and MD-2 (3, 8, 11–15). The transduction mechanism for TLR4 activation is well characterized for LPS from gram negative bacteria. CD14 delivers LPS to MD-2, which forms a stable heterodimer with the extracellular domain of TLR4 (16), leading to dimerization of the TLR4-MD-2 complex (17). This brings the toll/interleukin-1 receptor (TIR) domains on the cytoplasmic tails of TLR4 together allowing recruitment of cytoplasmic adapter molecules that promote oxidant production by NADPH oxidase and activation of downstream signaling pathways, leading to activation of pro-inflammatory transcription factors, such as NF-κB and AP-1, as well as the production of type 1 interferons (18).

MD-2 is co-expressed with TLR4 on the cell membrane of various cell types including leukocytes and endothelium (11, 19, 20), but soluble MD-2 (sMD-2) is elevated in plasma from patients with inflammatory conditions, such as sepsis, HIV infection, and endotoxemia (21–23). Epithelial cells in the gut and the airways express TLR4, but not MD-2, and are therefore entirely reliant on sMD-2 for TLR4 signaling (24–26). sMD-2 circulates in plasma of healthy individuals primarily as disulfide-linked oligomers (27). During experimental human endotoxemia, sMD-2 in septic plasma increases rapidly like an acute phase reactant and contains both sMD-2 oligomers and monomers. The monomeric form of sMD-2 represents the biologically active form of MD-2 (28).

In addition to being a required co-factor for TLR4 responses to LPS, MD-2 is also a required cofactor for TLR4 responses to heme (15). Heme binds to MD-2 at amino acids W23 and Y34 on MD-2, at a site independent of the LPS-binding site, to activate TLR4 pro-inflammatory signaling (15). Since SCD is a pro-inflammatory condition with an activated endothelium and elevated plasma heme, we examined sMD-2 levels in SCD plasma and its potential role in endothelial cell activation. We tested the hypothesis that sMD-2 is increased in SCD plasma and, by binding heme, contributes to pro-inflammatory signaling by endothelial cells.

## Materials and Methods

### Collection of Human and Mouse Blood

Human EDTA blood was obtained from healthy adult volunteers and SCD patients in steady-state after informed consent and according to protocols approved by the University of Minnesota’s Institutional Review Board in accordance with the Declaration of Helsinki. Mouse EDTA blood was collected from the inferior vena cava from homozygous Townes mice (29) expressing human HbS or HbA in accordance with protocols approved by University of Minnesota’s Institutional Animal Care and Use Committee. Human and mouse platelet-free plasma was stored at -85°C before use.

### sMD-2 ELISA

Human MD-2 ELISA kit (Millipore) was used to measure sMD-2 levels in human normal control and SCD plasma following the manufacturer’s instructions.

### sMD-2 Western Blots

Plasma samples were diluted with PBS (1:2 dilution for mouse plasma, and 1:5 dilution for human plasma). 2 µl of the diluted plasma was separated under denaturing conditions on 4-15% SDS-PAGE (Bio-Rad) and transferred to PVDF membranes (Millipore). Membranes were incubated with MD-2 primary antibody (Abcam) and fluorescent secondary antibodies (LI-COR). Fluorescence was quantitated with an Odyssey Image System (LI-COR). An IgG western blot or a total protein stain using AzureRed Fluorescent Total Protein Stain (Azure Biosystems) was used as a loading control.

### Endothelial IL-8, IL-6, and Soluble VCAM-1 Secretion

HUVEC were isolated and cultured as described previously (30). Human lung microvascular endothelial cells (HMVEC-L, Lonza) were cultured with microvascular endothelial cell growth medium-2 (Lonza) with 10% FBS. HUVEC or HMVEC-L in 24 well plates were treated with 2% of human AA or SS plasma in RPMI-1640 for 18 hours. IL-8, IL-6, and sVCAM-1 in the medium was measured using IL-8 and IL-6 ELISA kits (BioLegend), and a human VCAM-1 Quantikine ELISA kit (R&D Systems).

### Expression and Purification of N-Flag-Tagged Recombinant hMD-2

pFlag-CMV1–hMD-2 (a gift from Dr. Doug Golenbock, Addgene #13028) was sub-cloned into a Caggs expression plasmid. Chinese Hamster Ovary (CHO) cells were transfected with pT2/Caggs-Flag-hMD-2 to produce recombinant sMD-2 as described (31). CHO cells were maintained in RPMI-1640 with L-glutamine (Gibco) plus 10% fetal bovine serum in 5% CO_2_ at 37°C. Sixteen T225 flasks of CHO cells were used for each purification. Cells were transfected with polyethylenimine (PEI, linear, MW 2500, Polysciences) using 4:1 PEI to DNA (w/w). After 18 hours, cells were changed to ProCHO-AT protein-free media (Lonza). After 2-4 days, conditioned media were collected and centrifuged at 6000g for 30 minutes at 4°C and filtered using a 0.22 μm Stercup vacuum filter (Corning). Media collected after two days were used to treat HUVECs; media collected after four days were applied to an anti-Flag M2-affinity-gel (Sigma-Aldrich) column for sMD-2 purification. MD-2 was eluted with Flag peptide and concentrated using 10k centrifugal filter units (Amicon). The purity and concentration of the protein was determined by using 4-15% SDS-PAGE. Recombinant hMD-2 was confirmed by Western blot.

### Heme-Agarose Pull-Down Assays

To determine the physical interaction between sMD-2 and heme, human SS plasma was incubated overnight at 4°C with heme-agarose or control agarose (Sigma-Aldrich). After incubation, agarose beads were pelleted by centrifugation, washed, subjected to SDS-PAGE, and anti-MD-2 Western blot (15).

### Depletion of sMD-2 From SS Plasma

Anti-hMD-2 antibody (ab24182, Abcam) was coupled to CNBr-Sepharose (600 μg/ml gel; Sigma-Aldrich). Human SS plasma was diluted 5-fold in PBS and incubated at 4°C for 18 hours with the antibody-conjugated Sepharose. MD-2-depleted plasma was collected after pelleting the antibody-coated Sepharose by centrifugation and the MD-2 depletion was confirmed by western blot.

### Detecting sMD-2-Heme Binding by UV/Vis Absorbance

UV/Vis absorption spectra (250–550 nm) of heme, sMD-2, and heme plus sMD-2 were measured using a Nanophotometer (Implen). Rat hemopexin (HPX) (Athens Research & Technology) or recombinant hFlag-HPX was used as a heme-binding control (15).

### Statistical Analysis

Results are presented as means ± standard deviation. Comparisons of multiple treatment groups were made using One-Way ANOVA with the Holm-Sidak multiple comparison test (Sigma Stat). Significance testing between 2 groups was performed using Student’s paired or unpaired t-test as appropriate. Statistical significance was p < 0.05.

## Results

### sMD-2 Is Increased in SCD Plasma

**Figure 1:**
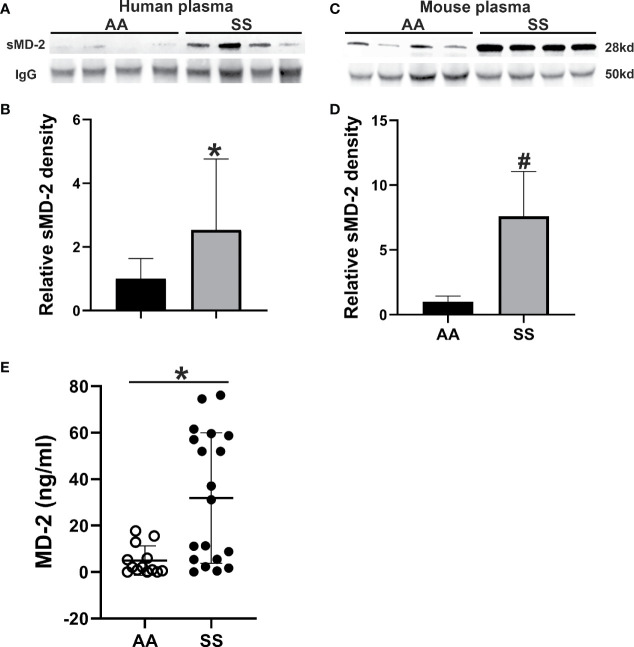
sMD-2 is increased in SCD plasma from humans and mice. **(A, C)** Representative western blots of sMD-2 from sickle (SS) and normal control (AA) plasma from humans and Townes mice. **(B, D)** Relative plasma sMD-2 on Western blots is calculated as the density ratios of sMD-2 bands compared to IgG in SS (n=17 for human, n=9 for mice) and AA (n=16 for human, n=7 for mice) plasma. **(E)** sMD-2 levels in human AA (n=13) and SS (n=19) plasma measured by ELISA. Bars are means ± SD, *p<0.02 and ^#^*p*<0.002.

As plasma sMD-2 is elevated in various inflammatory conditions and SCD is pro-inflammatory, we assessed plasma levels of sMD-2 in sickle (SS) and normal (AA) humans and mice by Western blot. sMD-2 was increased by 2.5-fold in human SS plasma compared to healthy AA plasma (p<0.02, **Figures 1A, B**). Similarly, in Townes SS mice, plasma sMD-2 was increased 7.6-fold compared to Townes control AA mice (p<0.002, **Figures 1C, D**). In addition, we measured human plasma sMD-2 level using an MD-2 ELISA kit. Mean sMD-2 levels were 35.6 ± 45.5 ng/ml in SS plasma, compared to 4.9 ± 6.3 ng/ml in AA control plasma (p<0.02, **Figure 1E**). The characteristics of human control and SCD subjects are summarized in **Supplementary Data Table 1**. Complete blood counts and serum chemistries of Townes AA and SS mice were published in a recent report from our lab (32).

### Endothelial Cells Secrete sMD-2 in Response to Heme

Potential sources of sMD-2 in plasma include the liver, monocyte-derived dendritic cells, and endothelial cells (22, 33, 34). LPS and TNF-α induce sMD-2 secretion by HUVEC (21, 22). To determine whether heme can induce sMD-2 secretion by endothelial cells, HUVEC were incubated with heme (0-30 µM) for 18 hours, and sMD-2 accumulation in the media was measured. sMD-2 in HUVEC culture media increased in a dose-responsive manner (**Supplemental Figure 1**). LPS (100 ng/ml), a positive control, also increased sMD-2 in HUVEC media.

### SCD Plasma Activates Endothelial Cells

**Figure 2:**
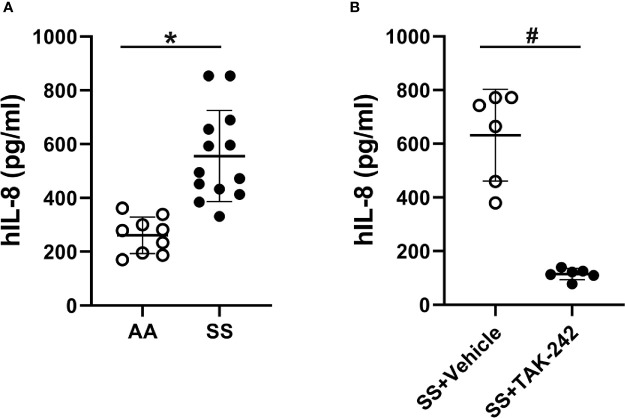
Human SS plasma induces IL-8 secretion in HUVEC through TLR4 signaling. **(A)** HUVEC were cultured with 2% AA (n=9) or SS (n=13) plasma in RPMI-1640 media for 18 hours. The IL-8 content in the conditioned medium was measured by ELISA. **(B)** HUVEC were cultured with 2% SS plasma plus vehicle or TLR4 signaling inhibitor, TAK-242 (1 µM) for 18 hours and IL-8 secretion into the media was measured by ELISA (n=6). Bars are means ± SD, *p<0.002, **^#^***p*<0.005.

SCD plasma contains a number of pro-inflammatory molecules (35–39). Platelets can contaminate plasma and release pro-inflammatory molecules into plasma that can activate endothelial cells. Therefore platelet-free plasma was used for these experiments to minimize their effects on endothelial cells. To determine whether SCD plasma could activate endothelial cells, we incubated HUVEC and HMVEC-L for 18 hours with media containing 2% AA or SS human platelet-free plasma and measured IL-8, IL-6 and sVCAM-1 in the media by ELISAs. IL-8 content in RPMI basal medium containing 2% AA or SS plasma before addition to HUVEC was too low to be measured by ELISA. IL-8 was 2-fold higher in the media of HUVEC treated with SS plasma (525.5 ± 167.0 pg/ml) compared to the media of HUVEC treated with AA plasma (260.9 ± 68.2 pg/ml, p<0.002, **Figure 2A**). In addition, sVCAM-1 was significantly increased in the media of HUVEC treated with SS plasma (13.42 ± 6.93 ng/ml) compared to HUVEC treated with AA plasma (5.48 ± 2.83 ng/ml, p<0.05, **Supplementary Figure S2A**). IL-6 was also significantly increased in the media of HUVEC treated with SS plasma (254.25 ± 33.18 pg/ml) compared to HUVEC treated with AA plasma (194.56 ± 46.04 pg/ml, p<0.005, **Supplementary Figure S2B**).

Like HUVEC, incubation of HMVEC-L with 2% SS plasma for 18 hours significantly increased IL-8, sVCAM-1 and IL-6 levels in the media compared to AA plasma (**Supplementary Figures S3A–C**). In subsequent experiments, we used IL-8 secretion by HUVEC to measure endothelial cell activation.

### SCD Plasma Activates TLR4 Signaling in Endothelial Cells

We have previously shown that heme binds to MD-2 and activates TLR4 signaling (3, 15). To determine whether SCD plasma activates endothelial cells through TLR4 signaling, we incubated HUVEC with media containing 2% SS or AA human plasma in the presence or absence of the TLR4 inhibitor TAK-242 and measured IL-8 in the media by ELISA. We found TAK-242 decreased IL-8 secretion induced by HUVEC incubated with SS plasma by 85% (p<0.005, **Figure 2B**). TAK-242 had no significant effects on IL-8 secretion in HUVEC incubated with AA plasma (145.1 ± 33.2 with TAK-242 and 152.1 ± 33. 8 without TAK-242, n=6). These results indicate that SS plasma increased IL-8 through TLR4 signaling.

### sMD-2 Contributes to SS Plasma-Induced IL-8 Secretion by HUVEC

**Figure 3:**
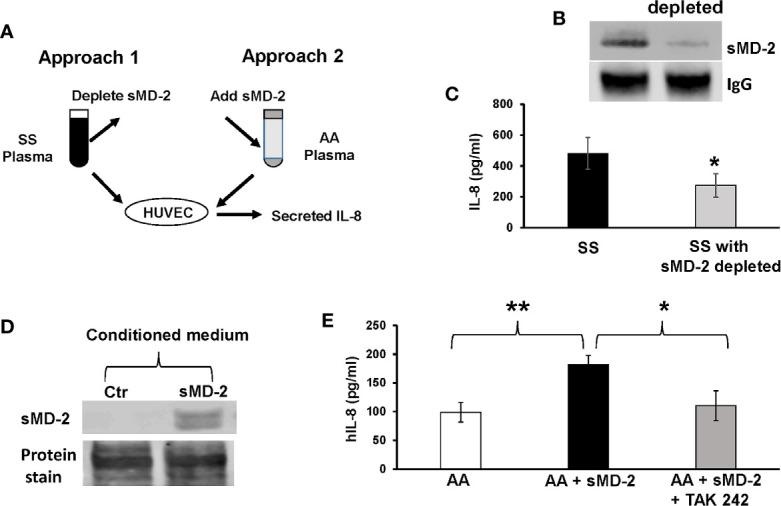
sMD-2 in plasma mediates IL-8 secretion by HUVEC. **(A)** Diagram shows two approaches to study sMD-2’s contribution to HUVEC IL-8 secretion. In approach 1, sMD-2 is depleted from SS plasma and in approach 2, sMD-2 is added to AA plasma. Plasma (2%) from both approaches were incubated with HUVEC for 18 hours and IL-8 secretion into the media was measured by ELISA. **(B)** Representative western blot of SS plasma after sMD-2 depletion. The IgG western blot was shown as loading controls. **(C)** IL-8 secretion by HUVEC after incubation with MD-2-depleted SS plasma. **(D)** Western blot of recombinant sMD-2 in conditioned medium from CHO cells transfected with MD-2-containing plasmids or non-transfected control CHO media. The total protein stain was shown as loading controls **(E)** sMD-2 CHO-conditioned medium or control CHO medium (10%) were added to HUVEC culture media along with 2% AA plasma in the presence or absence of TLR4 inhibitor, TAK-242 (1 µM), and incubated with HUVEC for 18 hours. The secreted IL-8 was measured by ELISA. Bars in C and E are means ± SD (n=3). *p<0.05, **p<0.01.

Plasma from SCD patients has many elevated pro-inflammatory molecules, such as IL-1β, IL-18, IL-6, and HMGB1 (35–39). Any of these could contribute to the increased IL-8 secretion by HUVEC incubated with SS plasma. To determine whether sMD-2 contributes to increased IL-8 secretion by HUVEC incubated with SS plasma, we used two approaches: depletion of sMD-2 from SS plasma and addition of sMD-2 to AA plasma (**Figure 3A**). Plasma samples from these two approaches were then incubated with HUVEC to determine their effects on IL-8 secretion. When sMD-2 was removed from SS plasma using an anti-MD-2 affinity column (**Figure 3B**), the sMD-2-depleted SS plasma reduced IL-8 secretion by 34.3% as compared to the same SS plasma without MD-2 depletion (p<0.05, **Figure 3C**). When conditioned CHO medium containing recombinant sMD-2 (**Figure 3D**) was added to AA plasma, the sMD-2-treated AA plasma increased IL-8 secretion by 1.8-fold (p<0.01, **Figure 3E**), and the TLR4 inhibitor, TAK-242, inhibited this increase (p<0.05, **Figure 3E**). Together, these results suggest that sMD-2 contributes to SS plasma-induced IL-8 secretion by HUVECs through TLR4 signaling.

### Polymyxin B Does Not Inhibit HUVEC IL-8 Secretion

Polymyxin B inhibits LPS-mediated TLR4 signaling (40). To determine whether the observed increased IL-8 secretion in HUVEC with SS plasma and recombinant sMD-2 was caused by LPS contamination, we added polymyxin B (Sigma-Aldrich) to our HUVEC culture media. LPS (10 ng/ml) induced IL-8 secretion in HUVEC that was inhibited by polymyxin B in a dose-responsive manner (**Supplemental Figure 4A**). Importantly, polymyxin B (1000 U/ml) did not inhibit IL-8 secretion in HUVEC treated with SS plasma or AA plasma + sMD-2 (**Supplemental Figures 4B, C**). Thus, HUVEC IL-8 secretion induced by SS plasma or AA plasma + sMD-2 was not mediated by LPS contamination.

### sMD-2 Binds Heme

**Figure 4:**
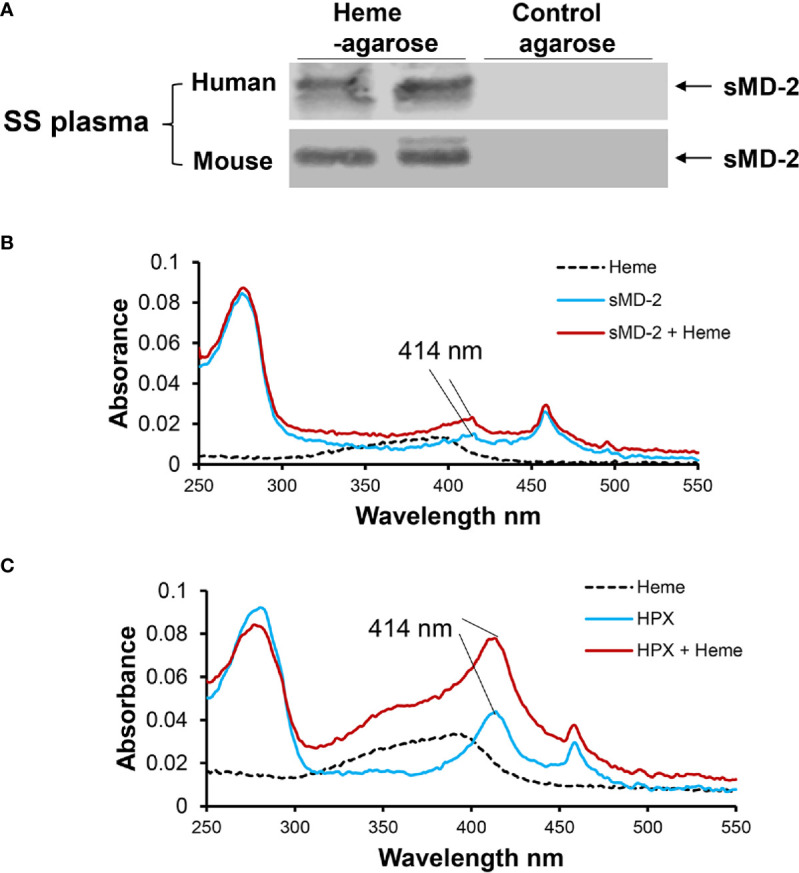
sMD-2 binds heme. **(A)** To determine the physical interaction between sMD-2 and heme, human SS plasma was incubated overnight at 4°C with heme-agarose or control agarose beads. The pelleted beads were washed with PBS 6 times. The pull-down proteins bound to the beads were then run on an SDS-PAGE Western bot using anti-MD-2 IgG as the primary detection antibody. Six SS plasmas were examined in independent experiments with similar results. Representative Western blots of 2 SS human and mouse plasmas are shown. **(B, C)** UV/Vis absorption spectra (250 - 550 nm) of recombinant **(B)** sMD-2 and **(C)** hemopexin (HPX, positive control), with and without added heme. Absorbance spectra show scans of heme alone (black dashed line), recombinant protein alone (blue line) and recombinant protein plus heme (red line). The Soret peak at 414 nm, indicative of heme binding, increases in the presence of added heme (red) for sMD-2 and HPX. In the absence of added heme, both recombinant proteins appear to have some bound heme (blue line) as shown by the Soret peak at 414 nm. The UV/Vis scans **(B, C)** are representative of 3 independent experiments.

We have previously shown that heme binds to MD-2 to initiate TLR4 signaling (15). We confirmed heme-binding to sMD-2 in plasma using heme-agarose pull-down followed by MD-2 Western blots. Heme-agarose or control agarose was incubated with SS plasma overnight, and then the agarose was pelleted by centrifugation. The proteins pulled-downed with the agarose beads were washed and eluted from the beads with SDS-containing buffer and run on MD-2 Western blots. Heme-agarose, but not control agarose, pulled down sMD-2 from SS human and mouse plasma (**Figure 4A**). To obtain additional evidence of heme binding to sMD-2, a standard heme–binding assay was performed as described previously (31). Heme binding to recombinant sMD-2 was assessed by scanning UV/Vis absorption spectrometry (250-550 nm, **Figure 4B**). Heme binding to recombinant hemopexin (HPX) was used as a positive control (**Figure 4C**). Absorbance spectra show scans of heme alone (black dashed line), recombinant protein alone (blue line), and recombinant protein plus heme (red line). The Soret peak at 414 nm, indicative of heme binding, increased in the presence of added heme (red line) for HPX and sMD-2. In the absence of added heme, both recombinant proteins appeared to have some bound heme as indicated by the Soret peak at 414 nm (blue line).

### Hemopexin Inhibits HUVEC IL-8 Secretion Induced by SS Plasma and AA Plasma Plus sMD-2

**Figure 5:**
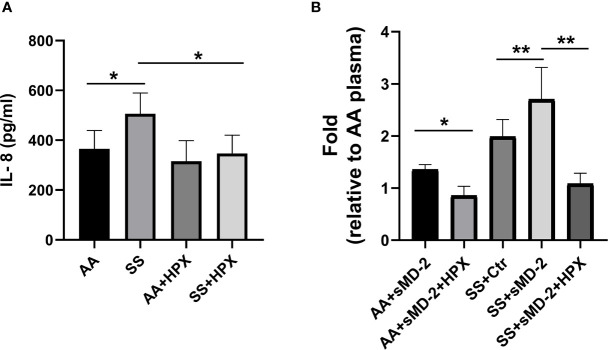
Hemopexin inhibits IL-8 secretion by HUVEC stimulated with human SS-plasma and AA-plasma plus sMD-2. **(A)** HUVEC media containing human AA or SS plasma (2%) was pre-incubated with recombinant hemopexin (HPX, 10 µM) for 30 minutes before being added to HUVEC for 18 hours. The IL-8 secreted into the HUVEC media was measured by ELISA (n=4). **(B)** CHO cells expressing recombinant MD-2 were cultured in protein-free medium for 2 days, the medium was collected as sMD-2-conditioned medium. HUVEC media containing AA (n=7) or SS (n=5) plasma (2%) plus sMD-2-conditioned medium (10%) was pre-incubated with HPX (10 µM) for 30 minutes before being added to HUVEC for 18 hours. The secreted IL-8 was measured by ELISA and presented as fold-change compared to AA-plasma +control conditioned medium without HPX. Bars are means ± SD. *p<0.05 and **p<0.01.

We have previously demonstrated that the high-affinity heme-binding protein hemopexin (HPX) blocks endothelial cell activation induced by heme-mediated TLR4 signaling (3). We examined whether heme plays a role in plasma sMD-2 stimulation of HUVEC IL-8 secretion. HPX has the highest heme-binding affinity (K_d_ ~10^-13^ M) among the known heme-binding proteins and can readily remove heme from other heme-binding proteins with lower heme affinity (41–43). When HPX was added to SS plasma, IL-8 secretion by HUVEC was reduced by 31.6% (p<0.05, **Figure 5A**). Adding HPX to AA plasma had no significant effect on IL-8 secretion. In addition, When HPX was added to AA or SS plasma plus recombinant sMD-2, HUVEC IL-8 secretion was significantly inhibited (AA p<0.05 and SS p<0.01, **Figure 5B**). Taken together, these results indicate that heme is necessary for enhanced HUVEC IL-8 secretion induced by SS plasma or AA plasma supplemented with sMD-2.

## Discussion

These data demonstrate that SCD plasma has increased levels of sMD-2 that can bind plasma heme and activate pro-inflammatory IL-8 secretion by endothelial cells through TLR4 signaling. The combination of heme-induced oxidative stress, inflammation, and adhesion of circulating blood cells to vascular endothelium is a key driver of the pro-inflammatory and prothrombotic vasculature that promotes sludging and stasis of blood flow in the post-capillary venules and ongoing ischemia-reperfusion physiology (44–46). Nature provides complicated systems to respond to heme overload, such as heme oxygenase-1 (HO-1) induction to degrade heme and heme-binding proteins to neutralize and transport heme (43, 47). Among the known heme-binding proteins, plasma HPX sequesters and transports heme to the liver for catabolism and detoxification *via* induction of HO-1 and ferritin (48, 49). Plasma HPX levels are depleted in SCD patients and mice because of chronic intravascular hemolysis (6, 7, 31). We have previously shown that increasing HPX through supplementation (3, 5) or gene transfer (31) prevents heme-induced inflammation and vaso-occlusion in SS mice. In this study, HPX-treated SS plasma lost its enhanced ability to induce IL-8 secretion in HUVEC, while adding HPX to AA plasma had no effect on basal IL-8 secretion. These findings demonstrate that heme can bind to sMD-2 and suggest that the addition of HPX to SS plasma sequesters heme away from sMD-2, thereby preventing TLR4-mediated IL-8 production by endothelial cells.

MD-2 expression can be regulated in specific tissue responses to sterile inflammatory stimuli and bacteria. In human hepatocytes, IL-6 induces the expression of MD-2 (34). In monocytes, corneal and intestinal epithelial cells, IL-10 or INF-γ can induce MD-2 expression (23, 50–52). Using immunohistochemical analysis, Wolfs et al. found MD-2 is expressed by endothelial cells and inflammatory cells in the livers and lungs of septic patients and suggested these cells are a source of the enhanced circulating sMD-2 levels during acute systemic inflammatory diseases such as endotoxemia and sepsis (22). In a recently published study, we found both MD-2 and MIP-2α (a murine IL-8 homologue) mRNA levels were increased in the livers of Townes SS mice compared to control AA mice, these differences are consistent with markedly increased hemolysis in Townes SS mice (32). In this study, we showed that HUVEC secreted sMD-2 in response to heme or LPS. We speculate that endothelial cells might be a potential source of some of the sMD-2 in SCD plasma, which will need further studies to validate.

MD-2 is a required accessory molecule for TLR4 signaling, indispensable for LPS recognition and signaling (25, 53). MD-2 knockout mice are hypo-responsive to LPS and are able to survive endotoxic shock, supporting a significant role of MD-2 in TLR4-dependent inflammatory responses *in vivo* (54). As a pattern recognition protein, MD-2 has been shown to bind to various ligands besides LPS to activate TLR4 signaling and promote inflammation, including heme (15), palmitic acid (55), angiotensin (56), and some synthetic compounds with no similarity to LPS (57). In our study, we found that heme agarose pulls-down sMD-2 from SCD plasma and recombinant sMD-2 has a Soret band at 414 nm, which indicates that sMD-2 binds heme.

Heme-induced microvascular stasis in SS mice requires endothelial TLR4 signaling (3). Heme activates TLR4 signaling on endothelial cells, leading to delivery of Weibel-Palade body constituents P-selectin and VWF to the surface of the vessel wall and the activation of the pro-inflammatory transcription factor NF-ĸB (3). Our finding that sMD-2-heme in SS plasma induces TLR4-dependent IL-8 secretion is in congruence with these previous observations. Thus, sMD-2 in plasma provides a potential pathway for heme-MD-2-mediated activation of TLR4 signaling to induce activation of the endothelium and vaso-occlusion in SCD.

The low level of IL-8, s-VCAM-1 and IL-6 production in HUVEC and HMVEC-L incubated with AA plasma and the incomplete inhibition of IL-8 secretion by HUVEC incubated with SS plasma depleted of sMD-2 or SS plasma plus TAK-242 indicates that other pro-inflammatory molecules in SS plasma might also induce HUVEC IL-8 secretion (35, 37, 39). A basal level TLR4 receptor activity induced by AA plasma has been reported before. Using a TLR4 reporter cell line that specifically recognizes ligands that bind and activate TLR4 (TLR4/NF-kB/SEAP Stable Reporter Cells), Xu et al. (39) found plasma from healthy AA control subjects induced low levels of TLR4 receptor activity; about 10% of the total TLR4 receptor activity induced by AA plasma was from HMGB1-dependent TLR4 receptor activity. In our study we found the TLR4 inhibitor TAK-242 had no significant effects on AA plasma-induced IL-8 secretion, suggesting other non-TLR4 pathways are likely contributing to the low level of IL-8 secretion induced by AA plasma. In SS plasma, 85% of IL-8 production by HUVEC was inhibited by TAK-242. The remaining IL-8 production induced by SS plasma could have been induced by other pro-inflammatory molecules that do not require TLR4 such as IL-1β, IL-18, and IL-6 (35–39).

The function of sMD-2 has been extensively studied in LPS-TLR4 signaling. Following synthesis, MD-2 is either secreted directly into the medium as a soluble, active protein, or binds directly to TLR4 in the endoplasmic reticulum before migrating to the cell surface (12). Heme activates TLR4 signaling by binding to a site on MD-2 that is independent and distinct from the LPS binding site on MD-2 (15). As shown in other studies (3, 8), heme-MD-2/TLR4-mediated pro-inflammatory effects are not due to LPS contamination. In this study, endothelial cell IL-8 secretion in response to SS plasma was not blocked by the LPS antagonist, polymyxin B.

SCD patients are known to be at higher risk for contracting bacterial infections compared to healthy humans (58, 59). The molecular pathophysiology that contributes to this infection susceptibility is incompletely understood. Because lung epithelial cells express TLR4 without MD-2, they remain resistant to endotoxin (25). sMD-2, produced either by neighboring cells or brought to the epithelial cells by plasma exudate, is required for LPS activation of lung epithelial cells (60). Increased circulating sMD-2 in SCD plasma could make SCD patients more susceptible to pulmonary infections and acute chest syndrome, especially in response to enhanced hemolysis (9, 61).

There are several limitations of this study. Although we found SS plasma increased IL-8, sVCAM-1, and IL-6 secretion in both HUVEC and HMVEC-L, endothelial cells vary widely among vascular beds such as lung and brain. The functions of sMD-2-heme in different endothelial cell beds and non-endothelial cells with TLR4 receptors, such as platelets and macrophages need be defined by separate studies.

In conclusion, SCD is considered a chronic inflammatory disease with hemolysis, vaso-occlusion, and ischemia-reperfusion, resulting in activation of the innate immune system and persistent activation of leukocytes, platelets, and endothelial cells. Therapeutic approaches for SCD include targeting HbS polymerization and the inflammatory processes that trigger vaso-occlusion (1, 35, 62). Clinical studies targeting TLR4 signaling as a therapeutic approach for sepsis and septic shock have brought several compounds and antibodies to clinical trials, with unsuccessful results. One such unsuccessful therapeutic is TAK-242, a small-molecule inhibitor of TLR4 signaling, that failed to suppress cytokine levels in patients with sepsis or respiratory failure in a phase III study (63). The LPS-MD-2/TLR4 antagonist, eritoran, also failed to reduce mortality among patients with severe sepsis (64). Nevertheless, targeting MD-2 to interfere with MD-2–TLR4 signaling has been extensively explored, and the results support the concept that MD-2 is an effective target to treat inflammatory disorders such as sepsis and acute lung injury (53, 65–68). We provide evidence that sMD-2 is increased in SCD, contributes to pro-inflammatory signaling in endothelial cells, and therefore, might be a potential therapeutic target for SCD and other hemolytic conditions.

## Data Availability Statement

The original contributions presented in the study are included in the article/**Supplementary Material**. Further inquiries can be directed to the corresponding author.

## Ethics Statement

The studies involving human participants were reviewed and approved by University of Minnesota’s Institutional Review Board. The patients/participants provided their written informed consent to participate in this study. The animal study was reviewed and approved by University of Minnesota’s Institutional Animal Care and Use Committee.

## Author Contributions

PZ, GMV, and JDBel designed the study, wrote, and edited the manuscript. PZ and JDBel prepared the figures. PZ, JN, and FA performed the experiments. JDBec and ATN collected data, analyzed results, and reviewed the manuscript. All authors contributed to the article and approved the submitted version.

## Funding

This work was funded by NIH grant R01 HL114567.

## Conflict of Interest

JDBec receives funding from Bayer not related to work herein. JDBel and GMV receive research funding from CSL Behring and Mitobridge (Astellas).

The remaining authors declare that the research was conducted in the absence of any commercial or financial relationships that could be construed as a potential conflict of interest.
