# Supplementary material for: Soluble MD-2 and Heme in Sickle Cell Disease Plasma Promote Pro-Inflammatory Signaling in Endothelial Cells
Source: Front Immunol. 2021 Mar 26;12:632709. doi: 10.3389/fimmu.2021.632709 (PMC8033004; doi:10.3389/fimmu.2021.632709)
Supplement: Supplementary file 1 [file DataSheet_1.pdf]

Supplementary Material

Table 1: Characteristics of Controls and Sickle Cell Subjects	
**Controls**	**N=13**
Age	NA
Gender	
Male	3
Female	10
Ethnicity	
Caucasian	9
Non-Caucasian	4
**Sickle Cell**	**N=19**
Age	33.7 (range 21-50)
Gender	
Male	9
Female	10
Genotype	
Hemoglobin S-S	12
Hemoglobin S-beta-thal	2
Hemoglobin S-C	5
CBC parameters†	
White blood cells (10^7/dL)	10.6 ± 0.8
Hemoglobin (g/dL)	8.8 ± 0.5
Hematocrit (%)	25 ± 1.5
Reticulocyte (absolute)	238 ± 38

†values mean ± standard error of means

Supplementary Table 1. Characteristics of controls and sickle cell disease subjects used in this study.

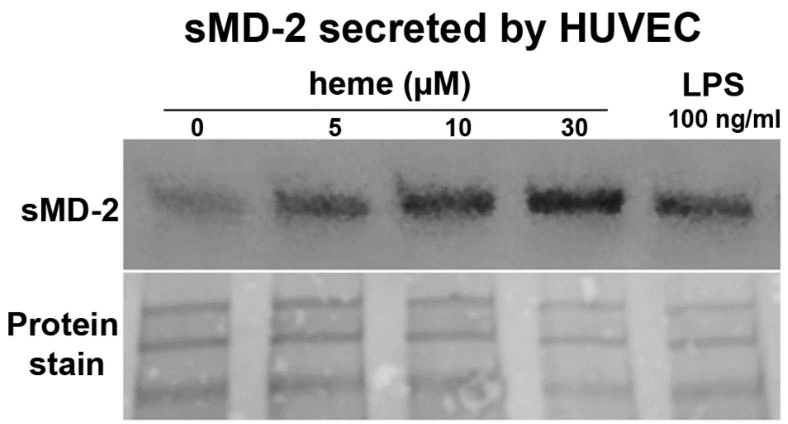

Supplementary Figure 1. Heme induces the secretion of sMD-2 by HUVEC. Heme (0-30 µM) was added to HUVEC cell culture medium (RPMI-1640 with 0.2% FBS) for 18 hours. The sMD-2 level in the medium was determined by Western blot. The loading controls are shown as total protein stain. LPS (100 ng/ml) was added to HUVEC culture medium as a positive control.

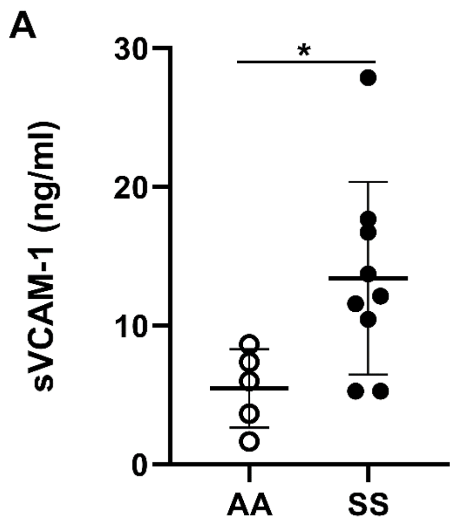

Supplementary Figure 2. Human SS plasma induces sVCAM-1 and IL-6 secretion in HUVEC. HUVEC were cultured with 2% AA or SS plasma in RPMI-1640 media for 18 hours. The sVCAM-1 (A) or IL-6 (B) content in the conditioned medium was measured by ELISA. Lines are means ± SD, *p<0.05, **p<0.005.

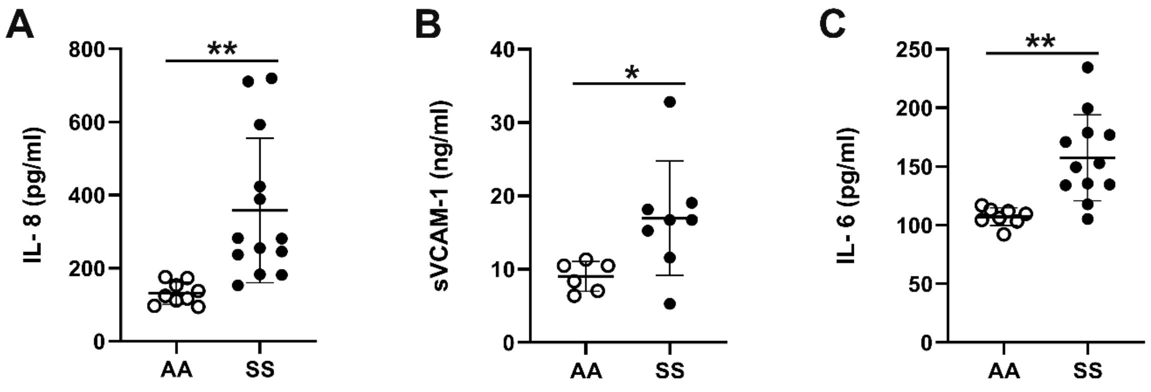

Supplementary Figure 3. Human SS plasma induces IL-8, sVCAM-1 and IL-6 secretion in HMVEC-L. Human lung microvascular endothelial cells (HMVEC-L) were cultured with 2% AA or SS plasma in RPMI-1640 media for 18 hours. The IL-8, sVCAM-1 and IL-6 content in the conditioned medium were measured by ELISA. (A) IL-8 was increased in SS plasma treated PMVEC-L (358.04 ± 197.71 pg/ml, n=13), compared to AA plasma treated HMVEC-L (131.57 ± 3.049 pg/ml, n= 9). (B) sVCAM-1 was increased in SS plasma treated HMVEC-L (16.95 ± 7.80 ng/ml, n=8) compared to AA plasma treated HMVEC-L (9.01 ± 2.04 ng/ml, n=6). (C) IL-6 was increased in SS plasma treated HMVEC-L (159.49 ± 37.56 pg/ml, n=12) compared to AA plasma treated HMVEC-L (107.86 ± 8.07 pg/ml, n=8). *p<0.05, **p<0.005.

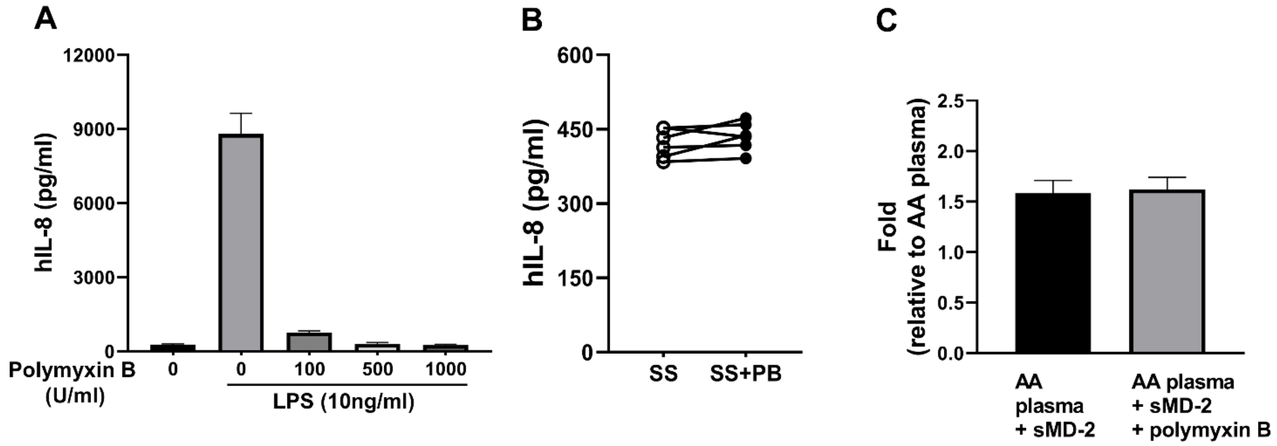

Supplementary Figure 4. LPS inhibitor Polymyxin B (PB) has no effects on SS plasma and sMD-2 induced IL-8 secretion in HUVEC. (A) HUVEC pretreated with various concentration of polymyxin B were stimulated with 10 ng/ml of LPS for 18 hours. (B) HUVEC were cultured with 2% SS plasma (n=6) or 2% SS plasma + polymyxin B (1000 U/ml) for 18 hours. (C) sMD-2 CHO-conditioned medium (10%) or control CHO medium plus 2% AA (n=4) plasma was added to HUVEC culture medium in the presence or absence of polymyxin B (1000 U/ml) for 18 hours culture. The secreted IL-8 content in the culture medium was measured by IL-8 ELISA.
